# Simultaneous Determination of Clidinium Bromide and Chlordiazepoxide in Combined Dosage Forms by High-Performance Liquid Chromatography

**DOI:** 10.1155/2013/417682

**Published:** 2013-02-24

**Authors:** Safwan Ashour, Nuha Kattan

**Affiliations:** Analytical Biochemistry Laboratory, Department of Chemistry, Faculty of Science, University of Aleppo, Aleppo, Syria

## Abstract

A sensitive and precise RP-HPLC method has been developed for the simultaneous estimation of clidinium bromide (CDB) and chlordiazepoxide (CDZ) in pure and pharmaceutical formulations. The separation was achieved on a Nucleodur C_8_ (250 × 4.6 mm i.d., 5 *μ*m particle size) column at 25°C. CH_3_CN-MeOH-NH_4_OAc 0.1M (30 : 40 : 30, v/v/v) was used as the mobile phase at a flow rate of 1.0 mL min^−1^ and detector wavelength at 218 nm. Almotriptan (ALT) was used as internal standard. The validation of the proposed method was carried out for linearity, accuracy, precision, LOD, LOQ, and robustness. The method showed good linearity in the ranges of 2.5–300.0 and 3.0–500.0 *μ*g mL^−1^ for CDB and CDZ, respectively. The percentage recovery obtained for CDB and CDZ was 100.40–103.38 and 99.98–105.59%, respectively. LOD and LOQ were 0.088 and 0.294 *μ*g mL^−1^ for CDB and 0.121 and 0.403 *μ*g mL^−1^ for CDZ, respectively. The proposed method was successfully applied to the determination of CDB and CDZ in combined dosage forms and the results tallied well with the label claim.

## 1. Introduction

Chlordiazepoxide (7-chloro-*N*-methyl-5-phenyl-3H-1, 4-benzodiazepine-2-amina-4-oxide) is used as an anxiolytic, sedative, hypnotic, anticonvulsant, and/or skeletal muscle relaxant. The drug may inhibit monosynaptic and polysynaptic reflexes by acting as an inhibitory neuronal transmitter or by blocking excitatory synaptic transmission. The drug may also directly depress motor nerve and muscle function [1, 2]. Clidinium bromide (3-[(hydroxy-diphenylacetyl)-oxy]-1-methyl-1-azoniabicylo-[2.2.2] octane bromide is an anticholinergic drug which may help symptoms of cramping and abdominal stomach pain by decreasing stomach acid and slowing the intestines. It is commonly prescribed in combination with chlordiazepoxide by the name of clidinium-c [3]. The United States Pharmacopeia (USP) stated the nonaqueous titration method for the assay of clidinium bromide and chlordiazepoxide [4]. Few methods for the determination of clidinium bromide and chlordiazepoxide in combined dosage forms including HPLC [5–7], derivative spectrophotometry [8, 9], spectrophotometry using multivariate calibration techniques [10], and capillary SFC [11] have been reported. Literature survey revealed that some analytical methods have been used for the individual estimation of clidinium bromide and chlordiazepoxide. Capillary electrophoresis [12] and kinetic spectrophotometric [13] methods for clidinium bromide have been described. Chlordiazepoxide has been determined either alone or with other compounds in pharmaceutical formulations using high-performance liquid chromatography [14–23], first-derivative spectrophotometry [17], spectrophotometry [23, 24], HPTLC [23, 25], voltammetry [26], and flow-injection potentiometry [27]. Several methods have been published for the determination of chlordiazepoxide in biological samples such as voltammetry [26], LC [28], and spectrophotometry [29]. In this work, a new reversed-phase high-performance liquid chromatographic method is proposed for the simultaneous determination of clidinium bromide and chlordiazepoxide in combined dosage forms.

## 2. Experimental

### 2.1. Equipment

A high-performance liquid chromatographic system consisted of Hitachi (Japan) Model L-2000 equipped with a binary pump (model L-2130, flow rate range of 0.000–9.999 mL min^−1^), degasser, and a column oven (model L-2350, temperature range of 1–85°C). All samples were injected (10 *μ*L) using a Hitachi L-2200 autosampler (injection volume range of 0.1–100 *μ*L). Elutions of all analytes were monitored at 218 nm by using a Hitachi L-2455 absorbance detector (190–900 nm) containing a quartz flow cell (10 mm path and 13 *μ*L volume). Each chromatogram was analyzed and integrated automatically using automation system software.

### 2.2. Materials and Chemicals

Working reference standards of clidinium bromide (CDB), chlordiazepoxide (CDZ), and almotriptan (ALT) were supplied by MSN Laboratories Ltd., Centaur Pharmaceuticals PVT. Ltd., and SMS Pharmaceuticals Ltd., (India), respectively. HPLC grade methanol, acetonitrile, and water were purchased from Labscan (Ireland) and analytical reagent grade ammonium acetate (Merck) was used to prepare the mobile phase. Tablets were purchased from Syrian market, containing clidinium bromide 2.5 mg and chlordiazepoxide 5 mg per tablet.

### 2.3. Chromatographic Conditions and Measurement Procedure

Chromatographic separation was performed on a reversed-phase Nucleodur column C_8_ (250 × 4.6 mm i.d., 5 *μ*m particle size) Macherey Nagle (Germany). The mobile phase was a mixture of acetonitrile : methanol : ammonium acetate (30 : 40 : 30, v/v/v). The mobile phase was filtered through a 0.45 *μ*m nylon-membrane filter and degassed by ultrasonic agitation prior to use. A flow rate of 1.0 mL min^−1^ was used in order to separate clidinium bromide, chlordiazepoxide and the internal standard almotriptan. The injection volume was 10 *μ*L. Peak areas were measured and HPLC analysis was conducted at ambient temperature.

### 2.4. Standard Solutions and Calibration Graphs

Individual stock standard solutions of clidinium bromide (2000.0 *μ*g mL^−1^) and chlordiazepoxide (2000.0 *μ*g mL^−1^) were prepared by dissolving appropriate amounts of pure drugs in methanol in separate brown volumetric flasks. These solutions were stored in the dark under refrigeration at 4°C and were found to be stable for ten days. A series of working standard solutions of CDB and CDZ were prepared by the appropriate dilution of the above mentioned stock standard solution in the methanol to reach concentration ranges of 2.5–300.0 and 3.0–500.0 *μ*g mL^−1^ for CDB and CDZ, respectively. In each sample 100 *μ*g mL^−1^ of the internal standard ALT was added. Working standard solutions were found to be stable during the analysis time.

To construct the calibration curve five replicates (10 *μ*L) of each standard solution were injected immediately after preparation into the column and the peak areas of the chromatograms were measured. Then, the mean peak area ratio of CDB and CDZ to that of the internal standard was plotted against the corresponding concentration to obtain the calibration graph.

### 2.5. Tablet (or Capsule) Sample Solutions

Twenty tablets were accurately weighted and finely pulverized. In the case of capsules, the contents of twenty capsules were completely evacuated from shells. An appropriate portion of this powder, equivalent to five tablets content of CDB and CDZ, was placed in a 25 mL volumetric flask with 20 mL of methanol. The solution was sonicated for 15 min and diluted to volume with methanol to obtain solution of CDB (500 *μ*g mL^−1^) and CDZ (1000 *μ*g mL^−1^). This sample solution was filtered using a 0.45 *μ*m nylon filter paper. Consequently a 3 mL aliquot of this solution was further diluted to 10 mL methanol containing 100 *μ*g mL^−1^ of the internal standard ALT; 10 *μ*L sample was injected into the HPLC system. Peak area ratios of CDB and CDZ to that of the internal standard were then measured for the determinations. These solutions were stored in the dark at 4°C and found to be stable for ten days at least.

### 2.6. Method Validation

The HPLC method was validated in terms of precision, accuracy, and linearity according to ICH guidelines. Assay method precision was determined using five independent test solutions. The intermediate precision of the assay method was also evaluated using different analysts on three different days. For intra day precision, different concentrations of CDB and CDZ were analyzed five times on the same day whereas for inter day precision the same drug concentrations were analyzed on three different days, and the percentage RSD of area was calculated. The accuracy of the assay method was evaluated with the recovery. Linearity test solutions were prepared as described in Section 2.4. Linearity was studied by injecting seven concentrations in replicates of five of the standard CDB and CDZ into the HPLC system. The peak area versus concentration data was performed by least-squares linear regression analysis. The limits of detection (LOD) and quantitation (LOQ) values were calculated. To determine the robustness of the method, the final experimental conditions were purposely altered and the results were examined. The flow rate varied by (±) 0.1 mL min^−1^, the percentage of organic modifier varied by (±) 5%, and pH of mobile phase varied by (±) 0.1.

## 3. Results and Discussion

### 3.1. Optimization of the Chromatographic Conditions

**Figure 1:**
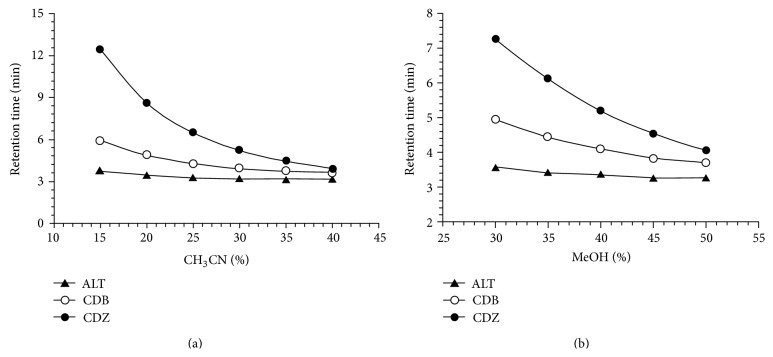
Plots of the retention time versus methanol or acetonitrile percentage in the mobile phase of ALT, CDB, and CDZ.

The optimized compositions were used for the analysis of all solutions individually as well as in combination. The mobile phase used initially was composed of ammonium acetate (0.1 M) and methanol. However, to achieve the optimum resolution, a small portion of acetonitrile was added in the mobile phase until obtaining good results. The chromatographic conditions were optimized for separation of drugs by varying methanol, strength of buffer solution, pH, proportion of acetonitrile, and flow rate. During the optimization of the method, different columns (Nucleodur C_8_, 250 × 4.6 mm, 5 *μ*m; Nucleodur C_18_  250 × 4.6 mm, 5 *μ*m; Hypersil Gold C_8_  250 × 4.6 mm, 5 *μ*m; ODS Hypersil C_18_  250 × 4.6 mm, 5 *μ*m) were tested. The chromatographic separation was achieved on Nucleodur C_8_, (250 × 4.6 mm, 5 *μ*m) column at 25°C. The peak shape of ALT, CDB, and CDZ was found to be symmetrical. The effect of composition of the mobile phase and flow rate on the retention time of ALT, CDB, and CDZ was investigated. The effect of methanol and acetonitrile percentage in the mobile phase is presented in Figure 1.

**Figure 2:**
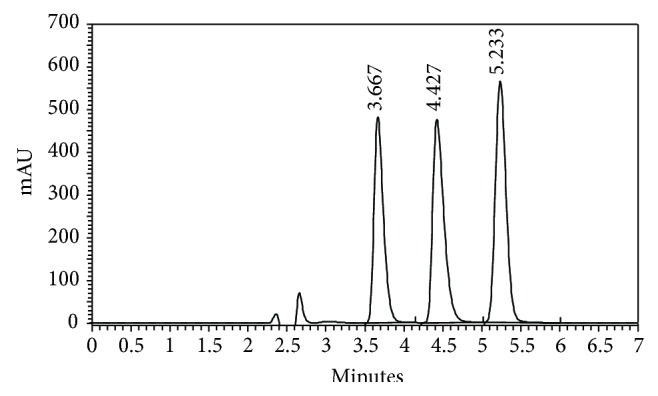
A typical chromatogram of a mixture of ALT (100 *μ*g mL^−1^), CDB (300 *μ*g mL^−1^), and CDZ (100 *μ*g mL^−1^) at retention times 3.667, 4.427, and 5.233 min, respectively. Chromatographic conditions: RP-HPLC on C_8_ column; mobile phase: acetonitrile-methanol-ammonium acetate 0.1 M (30 : 40 : 30, v/v/v); flow rate 1.0 mL min^−1^ and detection at 218 nm.

An increase in the percentage of methanol and acetonitrile decreases the retention of compounds, ALT, CDB, and CDZ. Increasing methanol percentage to more than 50% CDB peak is eluted with the solvent front, while at methanol percentage lower than 35% the elution of CDZ peak is seriously delayed. Also increasing acetonitrile percentage to more than 35% CDB peak is eluted with the solvent front, while at acetonitrile percentage lower than 20% the elution of CDZ peak is seriously delayed. The effect of pH in the chromatographic elution of the compounds was also investigated by changes the pH values of the aqueous component of the mobile phase from 4.0 to 6.0. A satisfactory separation and peak asymmetry for the drugs was obtained with mobile phase consisting of ammonium acetate (0.1 M, pH 5.0 adjusted with acetic acid)-methanol-acetonitrile (30 : 40 : 30, v/v/v), pumped at a flow rate 1.0 mL min^−1^ at 25°C. Quantitation was achieved with UV detection at 218 nm based on peak area. A representative chromatogram is shown in Figure 2. The retention times of ALT, CDB, and CDZ were 3.667, 4.427, and 5.233 min, respectively. 

### 3.2. Method Validation

#### 3.2.1. Selectivity

**Figure 3:**
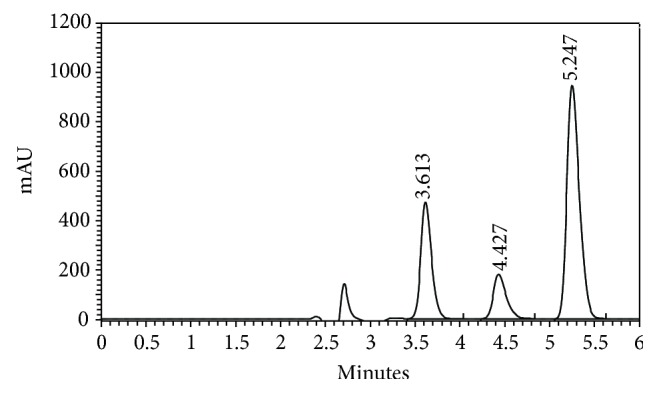
A typical chromatogram of a mixture of ALT (100 *μ*g mL^−1^), CDB (150 *μ*g mL^−1^), and CDZ (300 *μ*g mL^−1^) in the mobile phase, prepared from Laberax tablets.

The selectivity of the HPLC method is illustrated in Figure 2 where complete separation of ALT, CDB, and CDZ was noticed. The HPLC chromatogram recorded for the analytes in tablet (Figure 3) showed almost no peaks within a retention time range of 15 min. The figures show that CDB and CDZ are clearly separated and the peaks of analytes were pure and the excipients in the formulation did not interfere with the analyte. Thus, the HPLC method presented in this study is selective for CDB and CDZ.

#### 3.2.2. System Suitability

In the system suitability tests, five replicate injections of freshly prepared working standard solutions of CDB (300.0 *μ*g mL^−1^) and CDZ (100.0 *μ*g mL^−1^) in the presence of 100.0 *μ*g mL^−1^ of internal standard were injected into the chromatograph, and the theoretical plates, resolution factor, tailing factor, capacity factor, and % relative standard deviation (% RSD) of peak areas were determined. The results (Table 1) obtained from system suitability tests are in agreement with the USP requirements. The variation in peak area among five replicate injections of CDB and CDZ standard solutions was very low.

#### 3.2.3. Linearity, Sensitivity, and Limits of Quantification and Detection

The calibration curves for CDB and CDZ were linear over the concentration range of 2.5–300.0 *μ*g mL^−1^ and 3.0–500.0 *μ*g mL^−1^, respectively. Correlation coefficients (*r* ^2^) of the regression equations were greater than 0.999. The minimum level at which the investigated compounds can be reliably detected (limit of detection, LOD) and quantified (limit of quantitation, LOQ) was determined experimentally (Table 2). The LOD was expressed as the concentration of drug that generated a response to three times of the signal-to-noise (S/N) ratio, and the LOQ was 10 times of the S/N ratio. The LOD of CDB and CDZ attained as defined by IUPAC [30], LOD_(*k*=3)_ = *k* × *S*_*a*_/*b* (where *b* is the slope of the calibration curve and *S*_*a*_ is the standard deviation of the intercept), were found to be 0.09 and 0.12 *μ*g mL^−1^, respectively. The LOQ was also attained according to the IUPAC definition, LOD_(*k*=10)_ = *k* × *S*_*a*_/*b*, and were found to be 0.32 and 0.40 *μ*g mL^−1^, respectively.

#### 3.2.4. Accuracy and Precision

The precision and accuracy of the method were evaluated by analysis of seven samples for drugs mixture. Intraday assay variation was evaluated by injecting these samples in replicates of five in the same day. Interday assay variation was evaluated by injecting these samples in replicates of five on 4 different days from 1 to 10 days after preparation (Table 3). The standard deviation, relative standard deviation, recovery, and relative percentage error of different amounts tested were determined, as recorded in Table 3. The accuracy of the method is indicated by the excellent recovery and the precision is supported by the low standard deviation. Therefore, it was concluded that the procedure gives acceptable accuracy and precision for the analytes.

#### 3.2.5. Robustness

The robustness of an analytical procedure is a measure of its capacity to remain unaffected by small, but deliberate, variations in method parameters and provides an indication of its reliability during normal usage. Robustness of the method was investigated under a variety of conditions including changes of pH of the mobile phase, flow rate, and percentage of acetonitrile and methanol in the mobile phase. The standard solution is injected in five replicates and sample solution of 100% concentration is prepared and injected in triplicate for every condition and % R.S.D. of assay was calculated for each condition. The degree of reproducibility of the results obtained as a result of small deliberate variations in the method parameters has proven that the method is robust (Table 4).

#### 3.2.6. Stability Studies

Stability studies were carried out at laboratory temperature for 10 days to find potential stability problems of the drug in the formulations. Samples were analyzed at intervals of 0, 1, 5, and 10 days. The results obtained are given in Table 5. The percent RSD values between subsequent readings gave an indication of the stability of the drug in the formulations.

### 3.3. Application of the Assay

The developed method was successfully applied to analyze CDB and CDZ in marketed tablet formulations. The assay results are shown below for the average of five determinations of the four tablets. The performance of the proposed methods was assessed by comparison with the official method [8]. Mean values were obtained with Student's *t*- and *F*-tests at 95% confidence limits for four degrees of freedom. The results showed comparable accuracy (*t*-test) and precision (*F*-test), since the calculated values of *t*- and *F*-tests were less than the theoretical data. The proposed procedures were applied to determine CDB and CDZ in their pharmaceutical formulations (Figure 3). The results in Table 6 indicate the high accuracy and precision. As can be seen from Table 6, the proposed methods have the advantages of being virtually free from interferences by excipients such as glucose, lactose, and starch or from common degradation products. The results obtained were compared statistically by the Student's *t*-test (for accuracy) and the variance ratio *F*-test (for precision) with those obtained by the official method for the samples of the same batch (Table 6). The values of *t*- and *F*-tests obtained at 95% confidence level did not exceed the theoretical tabulated values indicating no significant difference between the methods compared.

## 4. Conclusion

A simple, specific, precise, and sensitive RP-HPLC method has been developed and validated for quantitative determination of clidinium bromide and chlordiazepoxide in raw materials and pharmaceutical preparations with a limit of detection of 0.088 and 0.121 *μ*g mL^−1^ for CDB and CDZ, respectively. The sample recoveries from all formulations were in good agreement with their respective label claims, which suggested noninterference of formulation excipients in the estimation. The developed method has more speed and higher sensitivity as compared to sophisticated spectrophotometric techniques and similar reported methods and has a wider range of linearity. Moreover, the lower solvent consumption along with the short analytical run time of 6.0 min leads to an environmentally friendly chromatographic procedure, which makes it especially suitable for routine quality control analysis work.

## Figures and Tables

**Table 1:** System suitability parameters.

Parameter	Almotriptan	Clidinium bromide	Chlordiazepoxide
Theoretical plates (*N*)	1711	4174	6598
Resolution factor^a^ (*R*_*s*_)	—	2.59	5.19
Tailing factor (*T*)	1.03	1.43	1.16
Capacity factor (*k*)	2.19	2.90	4.20
% RSD for seven injections	0.31	0.28	0.27

^a^ The resolution factor is calculated between each peak and its nearest preceding neighbor.

**Table 2:** Sensitivity and regression parameters.

Parameter	Clidinium bromide	Chlordiazepoxide
Optimum concentration range (*μ*g mL^−1^)	2.5–300.0	3.0–500.0
Regression equation∗	*A*_CDB_ = 0.4592*C*_CDB_ + 1.1366	*A*_CDZ_ = 1.1895*C*_CDZ_ + 2.2553
Correlation coefficient (*n* = 5)	0.9999	0.9999
Standard deviation of slope	0.0019	0.0025
Standard deviation of intercept	0.0135	0.0504
Regression equation∗∗	*R*_CDB/ALT_ = 0.003*C*_CDB_ + 0.009	*R*_CDZ/ALT_ = 0.009*C*_CDZ_ + 0.017
Correlation coefficient (*n* = 5)	0.9999	0.9999
Standard deviation of slope	1.1 × 10^−5^	2.8 × 10^−5^
Standard deviation of intercept	5.9 × 10^−4^	8.1 × 10^−4^
Limit of quantification, LOQ (*μ*g mL^−1^)	0.294	0.403
Limit of detection, LOD (*μ*g mL^−1^)	0.088	0.121

^*^ Regression equation for the peak area of drug versus concentration of drug in *μ*g mL^−1^.

∗∗Regression equation for the ratio of peak area of drug to that of I.S. versus concentration of drug in *μ*g mL^−1^.

**Table 3:** Accuracy and precision of within- and between-run analysis for the determination of clidinium bromide and chlordiazepoxide by HPLC.

Nominal concentration (*μ*g mL^−1^)	Intra-day (*n* = 5)			Inter-day (*n* = 5)		
	Mean ± SD *μ*g mL^−1^	RSD (%)	Recovery (%)	Mean ± SD *μ*g mL^−1^	RSD (%)	Recovery (%)
	Clidinium bromide					

2.50	2.52 ± 0.08	3.17	100.80	2.51 ± 0.07	2.79	100.40
6.50	6.68 ± 0.14	2.09	102.77	6.72 ± 0.17	2.53	103.38
25.00	25.63 ± 0.51	1.99	102.52	25.74 ± 0.28	1.09	102.96
50.00	50.86 ± 0.75	1.47	101.72	50.61 ± 0.39	0.77	101.22
75.00	75.78 ± 0.96	1.27	101.04	76.13 ± 0.43	0.56	101.51
150.00	150.92 ± 0.91	0.60	100.61	151.68 ± 0.75	0.49	101.12
300.00	301.92 ± 0.44	0.14	100.64	302.78 ± 1.73	0.57	100.93

	Chlordiazepoxide					

3.00	3.06 ± 0.09	2.94	102.00	3.04 ± 0.08	2.63	101.33
12.00	12.13 ± 0.14	1.15	101.08	12.10 ± 0.24	1.98	100.83
25.00	25.12 ± 0.23	0.92	100.48	25.20 ± 0.29	1.15	100.80
60.00	60.58 ± 0.54	0.89	100.96	60.98 ± 0.58	0.95	101.63
125.00	125.05 ± 0.44	0.35	100.04	125.42 ± 0.66	0.53	100.34
250.00	250.08 ± 0.56	0.22	100.03	249.96 ± 0.87	0.35	99.98
500.00	527.96 ± 0.97	0.18	105.59	519.83 ± 1.09	0.21	103.97

**Table 4:** Results of robustness study.

Factor	Level	Mean % assay (*n* = 3)		% RSD of results	
		Clidinium bromide	Chlordiazepoxide	Clidinium bromide	Chlordiazepoxide
pH of mobile phase	5.1	100.3	100.5	1.32	1.25
	4.9	100.1	100.7	0.86	0.69
Flow rate (mL/min)	0.9	99.9	100.1	0.97	1.04
	1.1	100.2	100.8	0.58	0.53
% of acetonitrile	25	99.7	100.04	1.24	0.95
	35	100.6	101.05	0.47	0.73
% of methanol	35	100.4	100.6	0.86	0.49
	45	100.6	100.9	0.79	0.61

**Table 5:** Stability study for the drug in different formulations.

Product^a^	Time (days)	Amount found^b^ (mg)		% Recovery		% ±RSD	
		CDB	CDZ	CDB	CDZ	CDB	CDZ
	0	2.51	5.04	100.40	100.80	1.91	0.22
Ribax capsules	1	2.52	5.05	100.80	101.00	0.98	0.49
	5	2.50	5.02	100.00	100.40	0.32	0.74
	10	2.49	5.01	99.60	100.20	1.05	0.86

	0	2.56	5.03	102.40	100.60	0.49	0.29
Laberax tablets	1	2.55	5.05	102.00	101.00	0.53	0.36
	5	2.51	5.04	100.40	100.80	0.82	0.40
	10	2.52	5.01	101.80	100.20	0.75	0.29

	0	2.58	5.01	103.20	100.20	1.13	0.31
Librax tablets	1	2.55	5.00	102.00	100.00	0.97	0.91
	5	2.53	4.99	101.20	99.80	0.68	0.87
	10	2.54	4.98	101.60	99.60	0.81	0.95

^a^ The dose is 2.5 mg CDB and 5.0 mg CDZ for all products.

^b^Five independent analyses.

**Table 6:** Determination of CDB and CDZ in pharmaceutical formulations by the proposed method and official method.

Sample	Clidinium bromide		Chlordiazepoxide	
	% Recovery^a^ ± S.D.			
	Proposed method	Official method	Proposed method	Official method
Ribax (2.5 mg CDB and 5.0 mg CDZ/capsule)				

*X* ± S.D.^a^	100.49 ± 1.92	100.97 ± 1.58	100.89 ± 0.22	100.04 ± 0.17
*t*-value^b^	1.96	2.03	2.12	1.89
*F*-value^b^	1.47		1.67	

Laberax (2.5 mg CDB and 5.0 mg CDZ/tablet)				

*X* ± S.D.^a^	102.58 ± 0.51	101.33 ± 0.42	100.59 ± 0.30	99.85 ± 0.23
*t*-value^b^	1.28	1.92	1.73	1.83
*F*-value^b^	1.47		1.70	

Librax (2.5 mg CDB and 5.0 mg CDZ/tablet)				

*X* ± S.D.^a^	103.45 ± 1.17	102.06 ± 1.08	100.25 ± 0.31	100.12 ± 0.28
*t*-value^b^	2.04	2.18	1.60	1.74
*F*-value^b^	1.17		1.23	

^a^Five independent analyses.

^b^Theoretical values for *t* and *F*-values at five degree of freedom and 95% confidence limit are (*t* = 2.776) and (*F* = 6.26).
